# Potential of Creatine in Glucose Management and Diabetes

**DOI:** 10.3390/nu13020570

**Published:** 2021-02-09

**Authors:** Marina Yazigi Solis, Guilherme Giannini Artioli, Bruno Gualano

**Affiliations:** Applied Physiology & Nutrition Research Group, School of Medicine, University of Sao Paulo, Sao Paulo 01246-903, Brazil; marina.solis@gmail.com (M.Y.S.); artioli@usp.br (G.G.A.)

**Keywords:** dietary supplements, exercise, skeletal muscle, glycemic control, type 2 diabetes mellitus

## Abstract

Creatine is one of the most popular supplements worldwide, and it is frequently used by both athletic and non-athletic populations to improve power, strength, muscle mass and performance. A growing body of evidence has been identified potential therapeutic effects of creatine in a wide variety of clinical conditions, such as cancer, muscle dystrophy and neurodegenerative disorders. Evidence has suggested that creatine supplementation alone, and mainly in combination with exercise training, may improve glucose metabolism in health individuals and insulin-resistant individuals, such as in those with type 2 diabetes mellitus. Creatine itself may stimulate insulin secretion in vitro, improve muscle glycogen stores and ameliorate hyperglycemia in animals. In addition, exercise induces numerous metabolic benefits, including increases in insulin-independent muscle glucose uptake and insulin sensitivity. It has been speculated that creatine supplementation combined with exercise training could result in additional improvements in glucose metabolism when compared with each intervention separately. The possible mechanism underlying the effects of combined exercise and creatine supplementation is an enhanced glucose transport into muscle cell by type 4 glucose transporter (GLUT-4) translocation to sarcolemma. Although preliminary findings from small-scale trials involving patients with type 2 diabetes mellitus are promising, the efficacy of creatine for improving glycemic control is yet to be confirmed. In this review, we aim to explore the possible therapeutic role of creatine supplementation on glucose management and as a potential anti-diabetic intervention, summarizing the current knowledge and highlighting the research gaps.

## 1. Introduction

Type 2 diabetes mellitus (T2DM) is a major public health concern worldwide, imposing high health costs for public and private health systems. According to the Global Burden of Disease Study, diabetes incidence increased from 11.3 million in 1990 to 22.9 million in 2017, whilst prevalence increased from 211.2 million in 1990 to 476.0 million in 2017 [1]. In 2017, the International Diabetes Federation estimated that 451 million adults live with diabetes, and by 2045, this number could increase to 693 million if no preventive measures are adopted [2].

T2DM is a metabolic disorder characterized by sustained hyperglycemia resulting from impaired insulin production by pancreatic β cells, impaired insulin action (i.e., insulin resistance), or both [3]. Chronic hyperglycemia in diabetes is associated with several cardiometabolic disorders, such as hypertension, dyslipidemia, atherosclerosis and visceral obesity. Moreover, T2DM is considered one of the top 10 causes of premature deaths from noncommunicable diseases, and is associated with increased mortality from infections, cardiovascular disease, stroke, chronic kidney disease, chronic liver disease, and cancer. In fact, all-cause mortality risk increases by 2- to 3-fold in individuals with diabetes [1].

T2DM can be managed with non-pharmacological treatment (i.e., weight reduction, dietary intervention, and physical activity) and/or pharmacological treatment [4]. There have been several dietary candidates to help control glycemia, so far with little or no clinical support from large, controlled trials. In the past two decades, creatine (α-methyl guanidine-acetic acid) supplementation has also been speculated as a dietary supplement potentially able to improve glucose control and insulin resistance.

Creatine is a naturally occurring amine, which is endogenously synthesized (~1 g∙d^−1^) in the liver, kidneys and pancreas from the amino acid glycine, methionine and arginine. Creatine can also be exogenously obtained from food sources (~1–5 g∙d^−1^), especially by the ingestion of beef, pork, chicken and fish. In humans, creatine is found in its free (60 to 70%) and phosphorylated (30 to 40%) forms. Approximately 95% of the total bodily creatine store is found in skeletal muscle, with the remaining 5% being found in cells with rapid energy demands, such as cardiac myocytes, retina, neurons and testicles. Creatine excretion occurs through its irreversible and non-enzymatic conversion to creatinine, which is then eliminated by the kidneys [5].

Creatine supplementation is a popular strategy to improve exercise performance in healthy individuals and athletes due to its efficacy of increasing muscle free creatine and phosphorylcreatine contents [6]. Strong evidence indicates that creatine supplementation increases muscle strength, lean mass and improve performance in high-intensity, short-duration exercise [7]. Moreover, new applications for creatine have been proposed, as creatine seems to have potential therapeutic properties in a wide variety of clinical conditions, such as muscle disorders, neurodegenerative conditions and, metabolic dysfunctions, including insulin resistance and T2DM [8].

There is preliminary evidence showing that creatine supplementation could affect glucose metabolism. Studies have demonstrated that creatine ingestion combined with carbohydrate promote greater total muscle glycogen accumulation in animals [9,10] and in humans [11]. Additionally, creatine supplementation along with carbohydrate promotes greater muscle creatine retention than creatine alone [12]. These effects may be partially explained by the fact that both muscle glucose and creatine uptake are influenced by insulin-dependent transporters. In vitro studies showed that creatine increases insulin secretion [13,14]; human studies, however, did not demonstrate the same effects [15,16]. In addition, creatine supplementation was shown to ameliorate hyperglycemia in transgenic Huntington mice and delay diabetes offset [17]. In humans, creatine supplementation associated with exercise training improved glycemia in sedentary [18] and T2DM adults [19]. Altogether, these findings provide the rationale for exploring the application of creatine as a potential anti-diabetic intervention. In this short, narrative review, we will describe the effects of creatine supplementation on glycemic control, summarizing the current knowledge and highlighting the research gaps.

## 2. Insulin resistance in the Context of the Interplay between Creatine and Glucose Metabolism

Under normal conditions, insulin is secreted by pancreatic β cells in response to the presence of energy substrates (e.g., glucose, fatty acids and amino acids), hormones and changes in energetic demands (e.g., fasting–feeding cycle, exercise and stress) in order to maintain normal blood glucose levels [3]. Insulin binds to its tyrosine kinase-type receptor and activates phosphorylation of a family of insulin receptor substrates (IRSs), especially IRS1 and IRS2 [20]. IRS-phosphorylated proteins bind and activate intracellular signaling molecules, such as phosphatidylinositol 3 kinase (PI3K), which in turn, promotes the translocation of the type 4 glucose transporter (GLUT-4) to the plasma membrane, ultimately resulting in the uptake of bloodstream glucose into the muscle skeletal [3]. Additionally, insulin stimulates the mitogen-activated protein kinase (MAPK) pathway, a necessary step in cell proliferation and nuclear activation [21].

Insulin resistance is a condition that precedes T2DM and, together with genetic and environmental factors, such as obesity and physical inactivity, may lead to the failure of β-cell function and, hence, a progressive decline in insulin secretion [22,23]. Insulin resistance is generally associated with suppressed PI3K pathway, with increased serine phosphorylation of IRS proteins and inhibited tyrosine phosphorylation [24]; IRS protein degradation also seems to occur in some conditions [25]. Additionally, insulin resistance in T2DM could display a suboptimal GLUT-4 translocation, irrespective of decreased muscle GLUT-4 content [19].

Recent evidence has suggested that T2DM individuals display altered creatine metabolism [26,27]. In a prospective cohort study including more than 4700 participants, Post et al. [27] observed that higher plasma creatine concentration was associated with increased incidence of T2DM. According to the authors, higher extracellular creatine concentration and lower intracellular phosphorylcreatine/creatine content may be related to an impaired intracellular energy state that suggests mitochondrial dysfunction, a postulated mechanism involved in T2DM pathophysiology [27]. Whether creatine is either a marker or maker in this process remains to be addressed.

**Figure 1:**
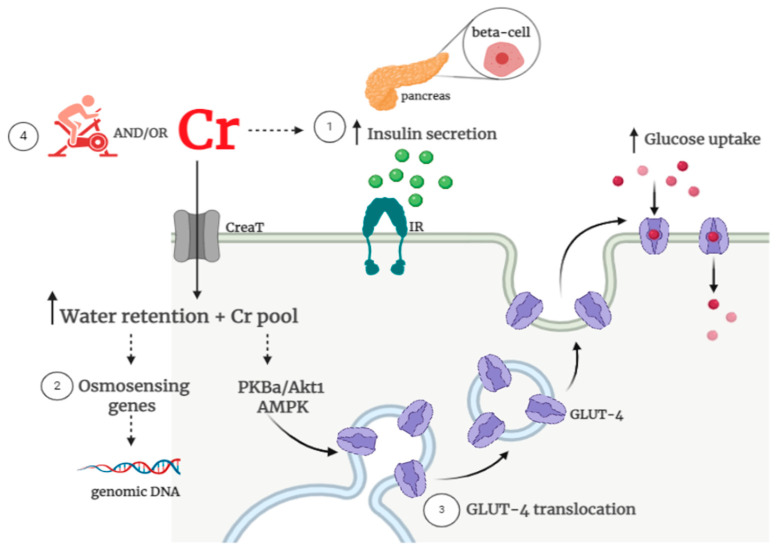
Possible creatine-related mechanisms on glycemic control. Potential mechanisms underpinning the role of creatine on glucose metabolism involve: (1) increased beta-cell insulin secretion; (2) improved water retention and osmosensing genes and (3) increased glucose uptake via type 4 glucose transporter (GLUT-4) content and activity. Additionally, (4) creatine supplementation could enhance the known benefits of exercise on glucose uptake/insulin sensitivity. There are currently insufficient clinical data to support all of these mechanisms. Note: Cr: creatine; CreaT: creatine transporter; IR: insulin receptor; GLUT-4: glucose transporter; PKBa/Akt1: protein kinase Bα; AMPK: Adenosine Monophosphate-activated protein kinase.

The potential basis for creatine supplementation to improve glycemic control involves: (1) creatine-induced increased insulin secretion; (2) creatine-induced changes in osmoregulation, and (3) creatine-induced increased glucose uptake via an augment in GLUT-4 content and/or translocation. In addition, exercise training has been suggested to have synergistic effects to creatine, leading to the assumption that these combined interventions could foster greater benefits in glycemic control vs. creatine or exercise alone [28]. The potential mechanisms that could explain the potential benefits of creatine, associated or not with exercise, on glucose control are illustrated in Figure 1.

In relation to the effects of creatine on insulin secretion, Hill et al. [29] were the first to show, in dogs, that an acute dose of creatine resulted in hypoglycemia. Later, in vitro studies confirmed that supraphysiological creatine concentrations elicited a modest stimulation of insulin secretion in isolated rat pancreas [14]. Similar results were shown in ex vivo experiments with mouse islets [13] and with insulinoma cells [30]. In pre-clinical studies involving different animal models, creatine supplementation increased circulating insulin levels [31] and improved insulinogenic index in T2DM rats [32]. However, human studies involving healthy [18] and T2DM adults [19] did not show increased insulin secretion either when creatine was provided alone or in combination with exercise training [19,33].

Creatine is also able to promote muscle water retention, thereby leading to changes in cell osmolarity [34]. Increased intracellular osmolarity induces cellular swelling, which may activate cell-volume sensitive signaling cascades capable of inducing adaptive changes in intra- and extracellular osmolarity. It is well documented that cell swelling is a potent stimulus to glycogen synthesis in muscle [35] and liver [36]. Thus, creatine-induced cell swelling could improve muscle glycogen stores. In fact, 10 days of creatine supplementation in health adults was able to increase glycogen stores and modulate mRNA content of genes and protein content involved in osmosensing, which could stimulate anabolic signal transduction, such as myogenin and, consequently, satellite cell activation [34]. In addition, increased intracellular osmolarity were associated with increases in circulating IGF-1, which promotes insulin-like effects and decreases counterregulatory hormones [37], which are involved in glycogen catabolism.

Some studies have suggested that oral creatine supplementation holds potential to promote muscle glucose uptake [9,19,38]. GLUT-4 is a key protein involved in transmembrane glucose transport and glucose uptake by skeletal muscle cells [39]. Increases in membrane GLUT-4 content could ultimately result in improved insulin sensitivity and glucose tolerance [9]. In rodents, creatine has been shown to increase *SLC2A4* (GLUT-4) gene expression and to enhance GLUT-4 protein content in muscle [10]. Similar effects have also been reported in humans after creatine supplementation accompanied by an exercise training program following limb immobilization [9], although not all studies have found the same outcomes [33,34]. Interestingly, creatine-induced improvements on glycemic control in T2DM patients were linked to increased GLUT-4 translocation to the sarcolemma, but not to changes in total muscle GLUT-4 content [19]. Increased GLUT-4 translocation was also associated with increased Adenosine Monophosphate-activated protein kinase (AMPK-α) expression, a protein involved in GLUT-4 translocation [38]. Likewise, among healthy young men, short-term creatine supplementation upregulated protein kinase Bα (PKBa/Akt1), a protein that plays a role in the insulin-stimulated GLUT-4 translocation and in glycogen synthesis [34].

Exercise knowingly modulates muscle glucose uptake by increasing (1) extracellular glucose delivery, from blood to muscle, (2) muscle membrane glucose transport, and (3) intracellular glucose phosphorylation and insulin sensitivity [40]. Importantly, T2DM patients retain the capacity of GLUT4 translocation to the sarcolemma in response to exercise [41], which makes exercise a potent means to improve glucose uptake. Exercise-induced GLUT-4 translocation seems to occur via AMPK pathway in an insulin-independent manner [42]. Thus, irrespective of circulating insulin levels or peripheral insulin action (which is facilitated trough muscle contraction via improvements in insulin signaling), exercise can directly induce a substantive GLUT-4 translocation to sarcolemma, thereby improving glycemic control [43]. Of relevance, preliminary evidence suggests that creatine has the potential to enhance the well-known beneficial effects of exercise on glucose control. The role of creatine supplementation, alone or in combination with exercise, on glucose metabolism is compressively covered in the next two subsections.

## 3. Effects of Creatine Supplementation Alone on Glycemic Control

Creatine supplementation increases intermuscular total creatine content by 10 to 30% in children, adults and older individuals [6,44]. Clinical evidence indicates that creatine supplementation improves fat-free mass [34], delays fatigue, increases muscle strength and, particularly in older adults, improves performance in activities of daily living [45,46,47]. Additionally, creatine supplementation upregulates genes and protein content of kinases involved in osmosensing and signal transduction, cytoskeleton remodeling, protein and glycogen synthesis regulation, satellite cell proliferation and differentiation [34]. Therefore, even without exercise, creatine alone seems to modulate skeletal muscle signaling, leading to potential short-term, physiological adaptations.

A study with animals revealed that 3 weeks of creatine administration (2% of the diet) significantly increased muscle glycogen stores possibly due to upregulation of *GLUT-4* expression and AMPK phosphorylation in female Wistar rats [10]. Moreover, Rooney et al. [31] showed increased plasma insulin concentrations in Wistar male rats supplemented with creatine (2% of the body weight) for eight weeks. The authors attributed this result to increased pancreatic total creatine content, which may have altered insulin secretion. In contrast, Op’t Eijnde et al. [9] reported that creatine intake (5 g∙kg^−1^∙d^−1^ of creatine for 5 days) in Wistar male rats did not increase GLUT-4 expression or enhance the sensitivity and the responsiveness of rat muscles to insulin. Similarly, Young and Young [48] did not find changes in glucose metabolism following creatine intake (300 mg∙kg^−1^∙d^−1^ of creatine over 5 weeks) in Sprague Dawley rats.

Creatine supplementation has been tested in different experimental paradigms of insulin resistance. Ferrante et al. [17] studied the effects of creatine ingestion in transgenic mice model of Huntington’s disease. Different doses of creatine (1, 2, or 3%) resulted in a substantial neuroprotective effect, improvement in the rotarod test performance and a significant reduction in hyperglycemia that typically accompanies this experimental model. Interestingly, the administration of creatine also delayed the onset of diabetes in these mice. In addition, Op’t Eijnde et al. [32] supplemented Goto-Kakizaki rats, a T2DM model, with creatine (2% of the diet) for eight weeks, and showed an improvement in the insulinogenic index (plasma glucose and insulin ratio), which was mostly attributed to a reduction in insulinemia. The authors concluded that creatine supplementation was able to improve insulin sensitivity in skeletal muscle of insulin-resistant rats. In contrast, using an animal model of severe muscle wasting and insulin resistance induced by high-dose dexamethasone, Nicastro et al. [49] showed that 7 days of creatine supplementation (5 g∙Kg^−1^∙d^−1^ of creatine) aggravated hyperglycemia and hyperinsulinemia in male Wistar rats. These findings highlight the complexities in interpreting and generalizing data obtained from creatine studies involving animal models, since there is a large specie-specific variability in response to creatine supplementation. This makes it difficult to reconcile pre-clinical data involving creatine [50]. The studies assessing the effect of creatine supplementation alone on glycemic control in animal models are summarized in Table 1.

In humans, creatine supplementation (5 g∙d^−1^ of creatine for 42 days) induced an increase in plasma glucose in response to an oral glucose load in health vegetarians adults [51]. Van Loon et al. [33] demonstrated that creatine supplementation alone (20 g∙d^−1^ for 5 days followed by 2 g∙d^−1^ for 6 weeks) increased glycogen content (+18%) in young, non-vegetarian adults. In contrast, Newman et al. [16] showed that creatine supplementation (20 g∙d^−1^ for 5 days followed by 3 g∙d^−1^ for 28 days) did not influence muscle glycogen content, plasma glucose and insulin responses during an oral glucose tolerance test in healthy, active, male adults. Additionally, insulin sensitivity surrogates (i.e., glucose-insulin index and index of insulin sensitivity) did not change with creatine ingestion, indicating that insulin action and secretion remained unaltered [16].

Van Loon et al. [33] showed no effect of creatine on *GLUT-4* mRNA and GLUT-4 protein content. Similarly, Safdar et al. [34] conducted a microarray analysis and did not observe changes in *GLUT-4* mRNA after creatine supplementation (20 g∙kg^−1^∙d^−1^ for 3 days followed by 5 mg∙kg^−1^∙d^−1^ for 7 days) in young, healthy men. Nevertheless, they observed an increase in *PKBa/Akt1* mRNA, which participates in glycogen synthesis via increasing glycogen synthase activity. The authors also showed a 21% decrease in skeletal muscle *phosphofructokinase* and *glycogen phosphorylase* mRNA, suggesting that creatine supplementation in the absence of exercise could eventually increase muscle glycogen stores by increasing the cascade signaling leading to GLUT-4 recruitment to the sarcolemma, despite the lack of changes in GLUT-4 [34]. In a significant different model, however, creatine supplementation (20 g∙d^−1^ for 2 weeks) was shown to prevent the drop in GLUT-4 protein expression induced by leg immobilization in healthy young men (+9% in the creatine group vs. −20% in the placebo group) [9]. In an open-label, cross-over study (with a 2-day washout period) involving a small sample of T2DM patients, creatine supplementation (6 g∙d^−1^ for 5 days) was as effective as metformin (2 × 500 mg∙d^−1^), a widely used anti-diabetic drug, in lowering blood glucose concentrations [52]. The human studies assessing the effect of creatine supplementation alone on glycemic control are summarized in Table 2.

## 4. Effects of Creatine Supplementation Combined with Exercise on Glycemic Control

Exercise has been recognized as a cornerstone in diabetes management, in addition to diet and hypoglycemic/antihyperglycemic agents. Regular physical activity induces beneficial metabolic and hemodynamic changes, resulting in improvements in insulin-independent muscle glucose uptake and in insulin sensitivity, as well as increased glycogen content [39,53]. The benefits of exercise as regard to glucose metabolism are widely reported in trained and untrained healthy [54], obese [55], insulin-resistant [56] and T2DM individuals [57]. Creatine supplementation has emerged as a strategy capable of enhancing the physiological and metabolic adaptations to exercise, which could confer protection in insulin resistance conditions [19,28,58]. Data supporting this hypothesis are so far inconclusive, however.

Souza et al. [59] showed that oral creatine administration (5 g∙kg^−1^∙d^−1^ for 7 days followed by 1 g∙kg^−1^∙d^−1^ for 8 weeks), associated or not with high-intensity swimming training, reduced plasma glucose levels in Wistar rats. Similarly, Araujo et al. [60] demonstrated that Wistar rats receiving creatine supplementation (diet supplemented with 13% for 7 days followed by 2% for 55 days) plus high-intensity exercise training exhibited a smaller area under the curve for glucose in response to an oral glucose challenge. On the other hand, Freire et al. [61] did not show any effect of creatine (2% of creatine in drinking water for 8 weeks) plus swimming training on plasma glucose responses to an oral glucose tolerance test in Wistar rats. Likewise, Vaisy et al. [62] did not demonstrate any benefits of creatine supplementation (diet enriched with 2.5% of creatine for 12 weeks) alone or in combination with exercise training on glucose homeostasis and muscular insulin sensitivity in Wistar rats with insulin resistance induced by a sucrose-rich cafeteria diet.

These conflicting results could be partially explained by the differences in creatine supplementation regimen and exercise types. For instance, both Freire et al. [61] and Vaisy et al. [62] used a single-phase creatine protocol throughout the entire intervention period, whereas others opted for a loading dose followed by a maintenance phase [62,63]. Training models also varied considerably with the use of treadmill [59,61,62] and swimming exercises [60], which precludes more direct comparisons concerning the exercise stimuli to which the animals were subjected. Therefore, the overall conclusions from pre-clinical studies are equivocal. The studies assessing the effects of creatine supplementation along exercise on glycemic control in animals are summarized in Table 3.

Op’t Eijnde et al. [9] provided new insights on the potential benefits of creatine plus exercise on glucose metabolism in humans. In this study, healthy individuals had one of their legs immobilized for two weeks, while they received either creatine (20 g∙d^−1^) or placebo supplementation. After the immobilization period, participants underwent a 10-week knee extension training (3 times a week). The immobilization period caused a significant reduction in GLUT-4 protein expression (−20%) in the control group, but not in the creatine supplemented group. The rehabilitation period promoted a normalization of the GLUT-4 content in the control group and a ~40% increase in GLUT-4 expression in the creatine group. Additionally, rehabilitation training per se did not increase muscle GLUT4 content above the baseline levels, but creatine along with training led to a substantial increase in this protein (+40%). Of relevance, the authors also reported that muscle glycogen was significantly augmented only when exercise training was undertaken in conjunction with creatine supplementation [9]. Using a similar experimental design, Derave et al. [63] showed that creatine (15 g∙d^−1^ during immobilization followed by 2.5 g∙d^−1^ for 6 weeks during rehabilitation) combined or not with protein supplementation was able to increase GLUT-4 protein expression and improve oral glucose tolerance following a 6-week exercise rehabilitation program that started two weeks after a single-leg immobilization protocol.

Gualano et al. [18] demonstrated that a 12-week creatine supplementation protocol (20 g∙d^−1^ for 10 days followed by 10 g∙d^−1^ throughout the remaining period) associated with moderate-intensity, aerobic exercise training (3 times a week) resulted in a greater decrease in plasma glucose in response to oral glucose tolerance test, compared to exercise alone, in sedentary but apparently healthy men. These data suggested a synergistic effect of exercise and creatine on glucose tolerance; however, in this and other studies [16,33], creatine did not change fasting insulin and Homeostatic Model Assessment for Insulin Resistance (HOMA-IR), a surrogate of insulin resistance.

Next to this preliminary study involving healthy participants, Gualano et al. [19] conducted a small-scale, double-blind, placebo-controlled trial involving T2DM patients, who underwent exercise training and received either creatine supplementation (5 g∙d^−1^ for 12 weeks) or placebo. In this study, creatine supplementation along with exercise training significantly reduced glycated hemoglobin (HbA1c) and glycemia in response to a meal tolerance test. No significant differences were observed for insulin and C-peptide concentrations. The beneficial effect on glycemic control was partially explained by an improvement in GLUT-4 translocation to sarcolemma, rather than in total muscle GLUT-4 content [19]. In an ancillary analysis from this study, decreased HbA1c levels and increased GLUT-4 translocation were associated with increased AMPK protein expression [38], providing new insights on the molecular mechanisms underlying the effects of combined creatine and exercise interventions on glucose metabolism. Of clinical relevance, creatine supplementation as an adjuvant therapy appeared to be safe, as no adverse events were reported and no alterations in health-related laboratory markers were seen [19,38].

However, the efficacy of creatine supplementation was not confirmed by a subsequent randomized, double-blind, placebo-controlled, pilot trial involving community-dwelling older adults [64]. After a 12-week follow-up period, creatine supplementation (5 g∙d^−1^) and resistance training (3 times a week) did not improve insulin resistance (assessed by fasting blood glucose and insulin, and HOMA-IR). It is possible to speculate that the better glycemic control exhibited by the participants in this study, compared to those of Gualano et al.’s study, may partially explain the null findings on the basis of a “ceiling effect”. The studies assessing the effect of creatine supplementation along exercise on glycemic control in humans are summarized in Table 4.

## 5. Conclusions and Future Directions

Creatine supplementation has the potential to promote changes in glucose metabolism that may favor a healthier metabolic profile. This may be particularly true when exercise training is provided along with this supplement, as creatine seems to enhance the training adaptations.

Evidence supporting the role of creatine on glucose metabolism remains speculative. As discussed, pre-clinical data are difficult to interpret as creatine responses are highly dependent on the experimental model adopted. The few existing clinical trials are small-scale, short-term and, therefore, exploratory. Despite the fact that a few of them have revealed promising benefits of creatine on glucose control, especially when exercise as co-prescribed, further large, longer-term, controlled trials involving T2DM with variable disease severity and under different pharmacological treatments are necessary to drawn firm conclusions on the efficacy and safety of creatine as an anti-diabetic intervention. It is equally important to develop new investigations aimed at unraveling the mechanisms by which creatine, aligned or not with training, could regulate glucose control, as basic research is indeed useful to better target potentially most benefited populations for testing creatine in next clinical trials.

## Figures and Tables

**Table 1:** Effect of creatine supplementation alone in glucose metabolism in animals.

Reference	Model	Creatine Protocol	Main Findings
Ferrante et al. [17]	Transgenic mice model of Huntington’s disease	Diet supplemented with 1, 2, or 3% of Cr for 21 d	↑ glucose tolerance; ↑ neuroprotective effect; ↑ body weight; ↑ motor performance on the rotarod test.
Op’t Eijnde et al. [9]	Male Wistar rats	Powdered rat chow with 5% of Cr for 5 d	↑ Cr and PCr muscle content; ↔ muscle GLUT-4 content; ↔ glucose transport rate; ↔ plasma insulin; ↔ blood glucose.
Young and Young, [48]	Male Sprague Dawley rats	300 mg∙kg^−1^∙d^−1^ for 5 wk	↑ Cr and PCr muscle content; ↔ basal rates of glucose uptake; ↔ insulin-stimulated rates of glucose uptake.
Rooney et al. [31]	Male Wister rats	Chow containing 2% of Cr for 2, 4, or 8 wk	↔ fasting plasma glucose; ↔ plasma glucose after oral glucose load; ↑ fasting plasma insulin levels; ↑ pancreatic TCr content.
Ju et al. [10]	Female Wistar rats	Chow containing 2% of Cr for 3 wk	↑ glycogen content; ↑ muscle GLUT-4 content; ↑ *GLUT-4* mRNA; ↑ AMPK phosphorylation; ↑ Acetyl-Coa carboxylase phosphorylation.
Op’t Eijnde et al. [32]	Male Goto-Kakizaki rats	Pallets enriched with 2% of Cr for 8 wk	↑ muscle Cr content only in young rats (but not in older rats); ↓ plasma insulin concentration; ↔ Blood D-glucose concentration after OGTT; ↓ insulinogenic index.
Nicastro et al. [49]	Male Wistar rats	5 g∙Kg^−1^∙d^−1^ of Cr for 7 d + 5 mg∙kg^−1^∙d^−1^ of DXM	↑ serum glucose and insulin after Cr + DXM; ↑ HOMA-IR after Cr + DXM; ↓ GLUT-4 translocation after Cr + DXM

Notes: ↑: increase; ↓: decrease; ↔: no change; Cr: Creatine; PCr: phosphorylcreatine; TCr: total creatine; OGTT: oral glucose tolerance test; GLUT-4: glucose transporter; AMPK: AMP-activated protein kinase; HOMA-IR: Homeostatic Model Assessment for Insulin Resistance (a surrogate of insulin resistance); DXM: dexamethasone.

**Table 2:** Effect of creatine supplementation alone in glucose metabolism in humans.

Reference	Sample *(n)*	Study Design	Creatine Protocol	Main Findings
Newman et al. [16]	Healthy, active, untrained, male adults (17)	Sigle-blind, placebo-controlled trial	Loading phase: 20 g∙d^−1^ (4 × 5 g) of Cr for 5 d + Maintenance phase: 3 g∙d^−1^ for 28 d	↑ muscle TCr; ↔ muscle glycogen content; ↔ plasma glucose and insulin during OGTT; ↔ glucose-insulin index; ↔ index of insulin sensitivity.
Rooney et al. [51]	Healthy, vegetarian adults (14)	Controlled-trial	5 g∙d^−1^ of Cr for 42 d	↑ plasma total Cr concentration; ↑ plasma glucose response; ↔ plasma insulin.
Van Loon et al. [33]	Young, nonvegetarians adults (20)	Double-blind placebo-controlled trial	Loading phase: 20 g∙d^−1^ (4 × 5 g) of Cr for 5 d + Maintenance phase: 2 g∙d^−1^ for 6 wk	↑ muscle glycogen, Cr and PCr after loading phase, with a decline in maintenance phase; ↔ *GLUT-4* mRNA; ↔ total muscle GLUT-4 protein content.
Safdar et al. [34]	Young, healthy, nonobese men (12)	Double-blind, crossover, randomized, placebo-controlled trial	Loading phase: 20 g∙d^−1^ (4 × 5 g) of Cr for 3 d + Maintenance phase: 5 g∙d^−1^ for 7 d	↑ muscle total Cr ↑ *PKB/Akt1* expression and protein; ↑ *MAPK* expression; ↔ *GLUT-4* mRNA
Rocic et al. [52]	Recently diagnosed T2DM patients, without anti-diabetic treatment (30)	Open-label, cross-over	6 g∙d^−1^ of Cr or 1000 mg∙d^−1^ of metformin for 5 d	↓ glucose concentration in both groups; ↔ insulin, C-peptide and, HbA1c.

Notes: ↑: increase; ↓: decrease; ↔: no change; Cr: Creatine; PCr: phosphorylcreatine; TCr: total creatine; GLUT-4: glucose transporter; OGTT: oral glucose tolerance test; HbA1c: glycosylated hemoglobin; Akt1: protein kinase B; MAPK: mitogen-activated protein kinases.

**Table 3:** Effect of creatine supplementation along with exercise in glucose metabolism in animals.

Reference	Model	Creatine and Training Protocol	Main Findings
Souza et al. [59]	Male Wistar rats	Loading phase: 5 g∙kg^−1^ body weight of Cr for 7 d + Maintenance phase: 1 g∙d^−1^ for 8 wk Training: swimming	↓ plasma glucose levels during 1–4 wk after Cr alone; ↓ plasma glucose levels during 1–8 wk after Cr and exercise protocol.
Freire et al. [61]	Male Wistar rats	Pallets enriched with 2% of Cr for 4 or 8 wk Training: swimming	↔ glucose uptake; ↔ glucose AUC during OGTT; ↔ liver and quadriceps glycogen content.
Vaisy et al. [62]	Male Wistar rats	Cafeteria diet enriched with 2.5% of Cr for 12 wk Training: swimming	↔ fasting blood glucose concentration; ↓ fasting plasma insulin level after training and training + creatine; ↓ whole body insulin level.
Araújo et al. [60]	Male Wistar rats	Loading phase: Chow containing 13% of Cr for 7d + Maintenance phase: Chow containing 2% of Cr for 55 d Training: high intensity treadmill running	↔ glucose uptake; ↓ glucose AUC during OGTT after Cr and exercise protocol.

Note: ↑: increase; ↓: decrease; ↔: no change; Cr: Creatine; PCr: phosphorylcreatine; TCr: total creatine; OGTT: oral glucose tolerance test; AUC: area under the curve.

**Table 4:** Effect of creatine supplementation along with exercise on glucose metabolism in humans.

Reference	Sample *(n)*	Study Design	Creatine and Training Protocol	Main Findings
Op’t Eijnde et al. [9]	Young, healthy subjects (22)	Double-blind placebo-controlled trial	Loading phase: 20 g∙d^−1^ during immobilization period (2 wk) + Maintenance phase: 15 g∙d^−1^ for 3 wk followed by 5 g∙d^−1^ for 7 wk during rehabilitation training Program training: knee-extensor resistance training (3 times∙wk^−1^)	Immobilization period: ↓ 20% GLUT-4 in placebo group, but not in Cr group; ↔ glycogen and Cr muscle content in both groups. Rehabilitation period: ↑ 40% GLUT-4 in Cr group; ↑ glycogen and Cr muscle content after Cr.
Derave et al. [63]	Young, healthy subjects (22)	Double-blind, placebo-controlled trial	Loading phase: 15 g∙d^−1^ during immobilization period (2 wk) combined or not with protein supplementation + Maintenance phase: 2.5 g∙d^−1^ for 6 wk during rehabilitation training Program training: knee-extensor resistance training (3 times∙wk^−1^)	Immobilization period: ↓ GLUT-4 in placebo and Cr group, but not in Cr + protein group; ↔ glycogen and Cr muscle content in all groups. Rehabilitation period: ↑ 24% GLUT-4 in Cr group and ↑ 33% in Cr + protein group; ↑ glycogen and Cr muscle content after Cr and Cr + protein supplementation.
Gualano et al. [18]	Healthy, sedentary male (22)	Double-blind, randomized-placebo-controlled trial	Loading phase: 0.3 g∙kg^−1^∙d^−1^ of Cr for 1 wk + Maintenance phase: 0.15 g∙kg^−1^∙d^−1^ for 11 wk Program training: aerobic training at 70% of the VO^2^max	↓ glucose AUC after OGTT; ↔ fasting insulin; ↔ HOMA-IR.
Gualano et al. [19]	T2DM patients (25)	Double-blind, randomized-placebo-controlled trial	5 g∙d^−1^ of Cr for 12 wk Program training: Aerobic training and resistance training	↓ HbA1c in Cr group; ↓ glycemia during MTT (0, 30 and 60 min) in Cr group; ↑ muscle PCr content in Cr group; ↑ muscle strength and function in Cr group
Alves et al. [38]	T2DM patients (25)	Double-blind, randomized-placebo-controlled trial	5 g∙d^−1^ of Cr for 12 wk Program training: Aerobic training and resistance training	↑ AMPK protein expression; ↔ IR-β, Akt1 and MAPK.
Oliveira et al. [64]	Healthy, older adults (32)	randomized, double-blind, placebo-controlled, parallel-group clinical trial	5 g∙d^−1^ of Cr for 12 wk Program training: resistance training	↔ inflammatory biomarkers ↔ fasting blood glucose; ↔ fasting insulin; ↔ HOMA-IR.

Note: ↑: increase; ↓: decrease; ↔: no change; Cr: Creatine; PCr: phosphorylcreatine; TCr: total creatine; OGTT: oral glucose tolerance test; GLUT-4: glucose transporter; T2DM: type 2 diabetes mellitus; AUC: area under the curve; HOMA-IR: Homeostatic Model Assessment for Insulin Resistance; HbA1c: glycosylated hemoglobin; IR-β: insulin receptor; Akt1: protein kinase B; MAPK: AMP-activated protein kinase.

## Data Availability

Not applicable.
